# TAFRO syndrome presenting as intrahepatic cholangitis on autopsy

**DOI:** 10.1002/ccr3.4005

**Published:** 2021-03-03

**Authors:** Hiroaki Nishioka, Shogo Nishino, Aisa Yoshizaki, Shigeo Hara

**Affiliations:** ^1^ Department of General Internal Medicine Kobe City Medical Center General Hospital Kobe Japan; ^2^ Department of Pathology Kobe City Medical Center General Hospital Kobe Japan

**Keywords:** alkaline phosphatase, Castleman disease, cholangitis, TAFRO syndrome

## Abstract

Elevation of ALP is an abnormal feature in TAFRO syndrome, but the cause is unknown. This article is the first report that histologically showed intrahepatic cholangitis may be the cause of ALP elevation in TAFRO syndrome.

## Graphical abstract

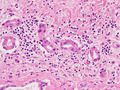

## 1 INTRODUCTION

TAFRO syndrome (thrombocytopenia, anasarca, myelofibrosis, renal dysfunction, and organomegaly) is an atypical manifestation of multicentric Castleman disease. Elevation of alkaline phosphatase (ALP) has also been reported to be an abnormal feature in TAFRO syndrome. Hepatic or biliary involvement has been suspected; however, the histological findings in liver have rarely been reported. Herein, we report a case of 69‐year‐old Japanese woman with TAFRO syndrome showing intrahepatic cholangitis on autopsy. The patient was refractory to immunosuppressive therapies including prednisolone, tocilizumab, and cyclosporine A, and died. ALP was elevated at the first visit and continued to increase. Autopsy revealed intrahepatic cholangitis. Our findings suggest that intrahepatic cholangitis may be the cause of ALP elevation in TAFRO syndrome.

TAFRO syndrome is a systemic inflammatory disorder characterized by thrombocytopenia (T), anasarca (A), fever (F), reticulin fibrosis, renal failure (R), and organomegaly (O).^1^, ^2^ The diagnostic criteria contains 3 major criteria: anasarca (pleural effusion, ascites, or general edema), thrombocytopenia (**≤**100 000/μL), and systemic inflammation (fever above 37.5℃ and/or serum C‐reactive protein (CRP) ≥2 mg/dL), and 4 minor criteria: MCD‐like findings on lymph node biopsy, reticulin myelofibrosis, and/or increased megakaryocytes in bone marrow, organomegaly, and renal insufficiency. The presence of all 3 major and at least 2 of the 4 minor criteria is necessary for the diagnosis of TAFRO syndrome.^3^ TAFRO syndrome was thought to be a variant of human herpesvirus 8 (HHV‐8)‐negative idiopathic multicentric Castleman disease (iMCD)^1^, ^4^; however, the pathophysiology of TAFRO syndrome has been considered to differ from that of iMCD.^5^, ^6^ In TAFRO syndrome, elevation of alkaline phosphatase (ALP) without an increase in transaminases has frequently been reported.^3^, ^5^ Hepatic or biliary involvement has been suspected; however, hepatic histological findings have rarely been reported. Herein, we describe a case of TAFRO syndrome showing intrahepatic cholangitis revealed by autopsy.

## 2 CASE

**FIGURE 1:**
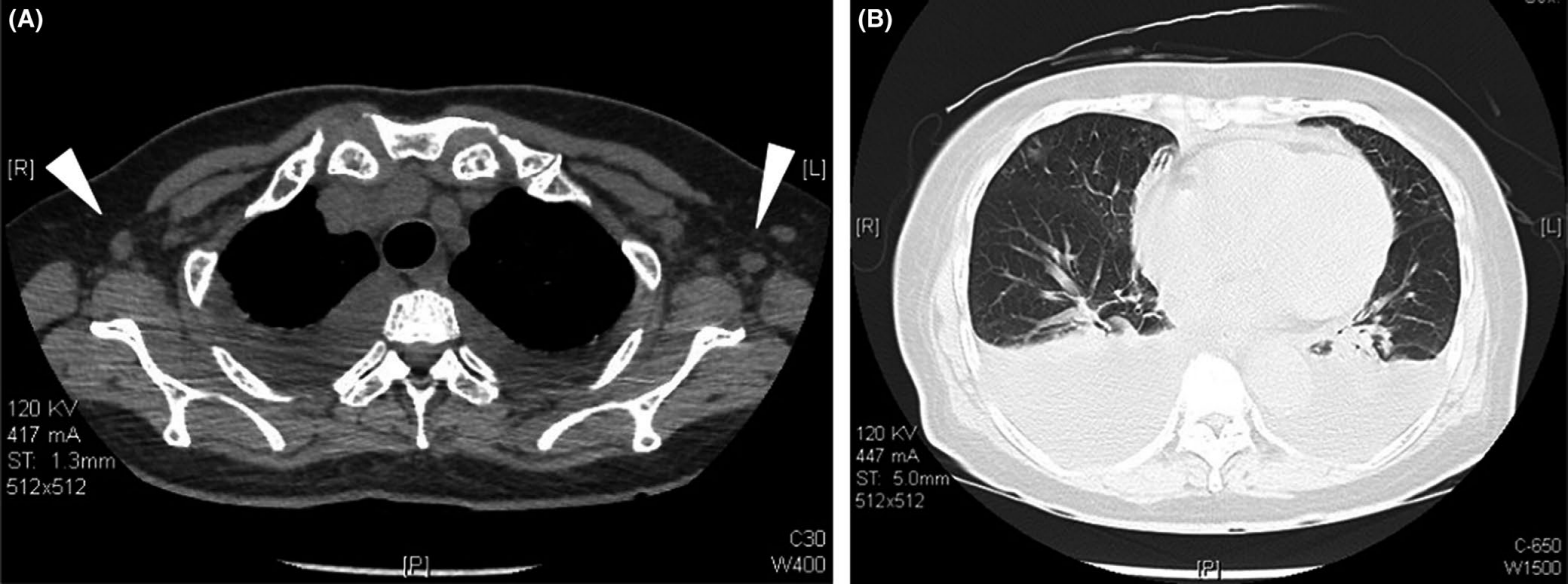
Chest computed tomography shows enlarged lymph nodes at the bilateral axillary area (white arrow heads) (A) and pleural effusion (B)

**FIGURE 2:**
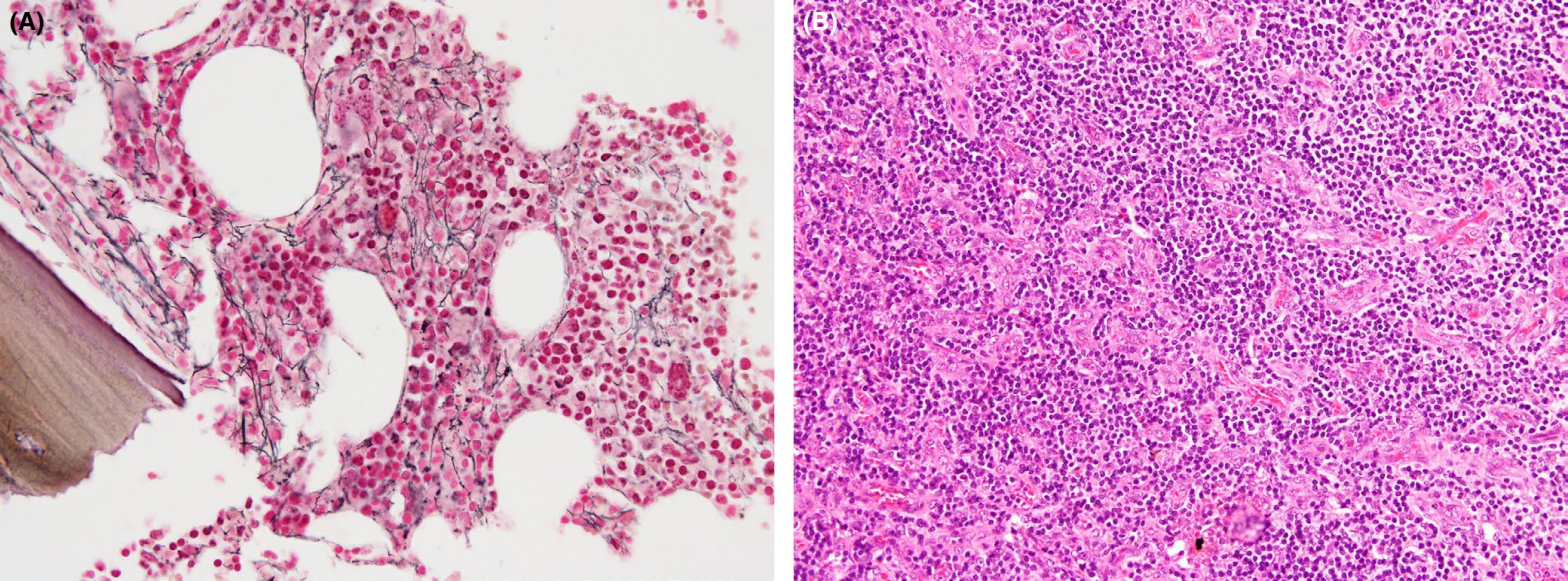
A, Silver staining of the bone marrow shows loose network of reticulin fibers (×400), corresponding to grade 1 myelofibrosis. B, Hematoxylin and eosin staining of the lymph node shows proliferation of dense endothelial venules in expanded interfollicular zone (×200)

A 69‐year‐old woman presented with a 10‐day history of fever and leg edema. Her medical history included hypertension without medication. On physical examination, the patient's blood pressure was 148/72 mm Hg; heart rate, 90 beats/minute; respiratory rate, 21 breaths/minute; and body temperature, 37.6°C. She was alert and conscious. Her abdomen was soft and distended. Pitting edema was observed on both legs. She did not show signs of polyneuropathy. Laboratory examination revealed white blood cell count of 5700/Μl, hemoglobin of 10.3 g/dL, platelet count of 61 000/μL, total protein of 5.1 g/dL, albumin of 2.1 g/dL, total bilirubin of 1.1 mg/dL, aspartate aminotransferase (AST) of 23 IU/L, alanine transaminase (ALT) of 14 IU/L, lactate dehydrogenase of 188 IU/L, ALP of 446 IU/L, γ‐glutamyltransferase (γ‐GTP) of 59 IU/L, blood urea nitrogen of 35.6 mg/dL, creatinine of 1.60 mg/dL, IgG of 992 mg/dL [normal range: 870‐1700], IgM of 87 mg/dL [35‐220], IgA of 206 mg/dL [110‐410], C3 of 92 mg/dL [65‐135], C4 of 20 mg/dL [13‐35], and CRP level of 15.5 mg/dL. Serum protein electrophoresis/immunofixation studies did not show any M‐protein. Antinuclear antibody, antineutrophil cytoplasmic antibodies, and antimitochondrial antibody were negative. Serum interleukin‐6 (IL‐6) was 47.5 pg/mL [reference range: 0‐2.4], and vascular endothelial growth factor (VEGF) was 1250 pg/mL [0‐38.3]. Urine analysis showed proteinuria of 0.6 g/day and hematuria with urinary casts. The serum human immunodeficiency virus (HIV) antigen and antibody test results were negative. Polymerase chain reaction for HHV‐8 in blood was negative. Chest and abdominal computed tomography showed enlarged lymph nodes at the bilateral axillary, mediastinal, and paraaortic areas; bilateral pleural effusion; ascites; and hepatosplenomegaly (Figure 1A,B). Abdominal ultrasonography showed enlarged liver with smooth surface and Doppler ultrasound did not detect any blood flow obstruction. Bone marrow histology showed normocellular and mild reticulin fibrosis, grade 1 (Figure 2A). Lymph nodes showed mild atrophic germinal centers and proliferation of highly dense endothelial venules in the medullary cord (Figure 2B). These symptoms and histological findings met the diagnostic criteria for TAFRO syndrome.

**FIGURE 3:**
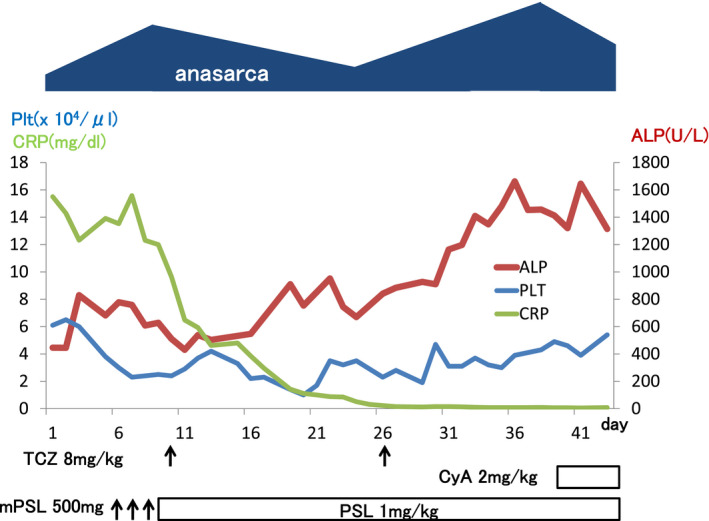
Clinical course of the patient. Plt, platelet; CRP, C‐reactive protein; ALP, alkaline phosphatase; TCZ, tocilizumab; CyA, cyclosporine A; mPSL, methylprednisolone

We intravenously administered methylprednisolone at 500 mg/day for 3 days (from days 6 to 8), followed by oral prednisolone at 60 mg/day, tocilizumab at 8 mg/kg every 2 weeks (days 10 and 26), and then cyclosporine A (2 mg/kg/day) continuously (from day 39). However, her fever and anasarca did not improve. The platelet count continued to decrease to 10 000/μL, ALP continued to increase to 1662 IU/l, and γ‐GTP increased to 913 IU/L. Total bilirubin slightly increased to 1.9 mg/dL, AST to 82 IU/L, and ALT to 94 IU/L. Creatinine remained at 1.69 mg/dL. On day 39, serum IL‐6 was 953 pg/mL and VEGF was 1490 pg/mL. The patient died on day 45 (Figure 3).

**FIGURE 4:**
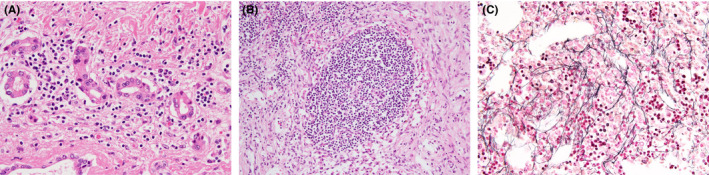
A, Hematoxylin and eosin staining of the liver revealed portal lymphocytic infiltration surrounding the bile duct (×400). B, Lymph node tissue showing atrophic follicles and vascular proliferation in the interfollicular space (×200). C, Bone marrow was hypercellular with grade 1 mild reticulin fibrosis (×400)

Autopsy revealed ascites (3550 mL) and liver swelling (1398 g). Enlargement of lymph nodes and spleen was not apparent. On gross examination, there was no thrombus in the portal and suprahepatic vein. Cut surface of the liver was smooth (Figure [Link], [Link]). Microscopically, hepatic histology revealed intraductal cholestasis and lymphocytic infiltration surrounding the bile duct, indicating intrahepatic cholangitis (Figure 4A). Interface and parenchymal inflammation was not apparent. Liver lobules showed no significant changes in the centrilobular region (Figure S2). Lymph node tissue showed atrophic follicles and vascular proliferation in the interfollicular space (Figure 4B). Bone marrow showed hypercellularity with grade 1 mild reticulin fibrosis (Figure 4C) and an increased number of megakaryocytes (not shown). In the kidney, glomeruli displayed mild endocapillary hypercellularity with endothelial cell swelling and mesangiolysis (not shown). The lungs revealed an organizing phase of diffuse alveolar damage that caused the patient's death.

## 3 DISCUSSION

TAFRO syndrome has been reported mostly in Japan, though it has recently been reported in other countries.^7^, ^8^, ^9^, ^10^ TAFRO syndrome was first defined as a novel systemic inflammatory disease at a consensus meeting of medical professionals and researchers in Japan in 2012.^1^ Masaki et al proposed a classification for diagnostic criteria and disease severity based on 28 cases of TAFRO syndrome in 2015.^3^ Our patient met the diagnostic criteria, satisfying all of the major and the minor criteria.

The differential diagnosis of TAFRO syndrome includes malignant lymphoma, multiple myeloma, IgG4‐related disease, systemic lupus erythematosus (SLE), HIV infection, and POEMS syndrome. In this case, histological findings of the lymph node and bone marrow did not show any malignant cells and lymphoplasmacytic infiltration. The patient's symptoms and laboratory data did not fulfill the criteria for the classification of SLE.^11^ Serum analysis showed negative results for HIV infection. POEMS syndrome was ruled out due to the absence of monoclonal gammopathy, skin changes, and polyneuropathy.

One additional feature that may support the diagnosis of TAFRO syndrome is ALP elevation.^2^, ^3^, ^5^ ALP is significantly higher in TAFRO syndrome than in iMCD.^5^ However, the mechanism of ALP elevation in TAFRO syndrome remains unclear. While several reports showed marked sinusoidal dilatation on liver biopsy in Castleman disease,^12^, ^13^ the histological findings in TAFRO syndrome are rarely described, probably because liver biopsy is difficult due to ascites and thrombocytopenia. Only one report written in Japanese showed cholangitis on liver biopsy.^14^ However, a few reports described nonspecific changes in the liver,^2^, ^15^, ^16^, ^17^ which might be modified by treatments. Herein, our patient was refractory to immunosuppressive therapies. ALP continuously increased, and intrahepatic cholangitis was discovered on autopsy. Our case suggests that intrahepatic cholangitis may cause ALP elevation in TAFRO syndrome.

Renal involvement is a common feature in TAFRO syndrome.^3^ However, the mechanism of renal dysfunction has not been clarified, because severe thrombocytopenia prevents kidney biopsy. A few biopsy reports describing the renal histology of TAFRO syndrome have been described; findings have included thrombotic microangiopathy‐like lesions^18^; membranoproliferative glomerulonephritis‐like appearance^19^; and diffuse endocapillary proliferation with mesangiolysis,^20^ which is similar to the autopsy results in our case.

In summary, we described an autopsy case of TAFRO syndrome with intrahepatic cholangitis. More case reports describing the liver histopathology will be necessary to elucidate the accurate mechanisms of ALP elevation with TAFRO syndrome.

## CONFLICT OF INTEREST

The authors declare that they have no conflict of interest.

## AUTHOR CONTRIBUTIONS

NH and AY: wrote the manuscript and made the literature review. SN and SH: provided support for creating pathological data. All the authors: read and approved the final manuscript.

## ETHICAL APPROVAL

The patient gave us her agreement to publish her clinical history, when she was alive. This case is anonymous.

## Supporting information

Fig S1A

Fig S1B

Fig S2

## Data Availability

Data sharing is not applicable to this article as no datasets were generated or analyzed during the current study.
