# Supplementary figures and images for: TAFRO syndrome presenting as intrahepatic cholangitis on autopsy
Source: Clin Case Rep. 2021 Mar 3;9(4):2254–8. doi: 10.1002/ccr3.4005 (PMC8077398; doi:10.1002/ccr3.4005)

## Slide 1
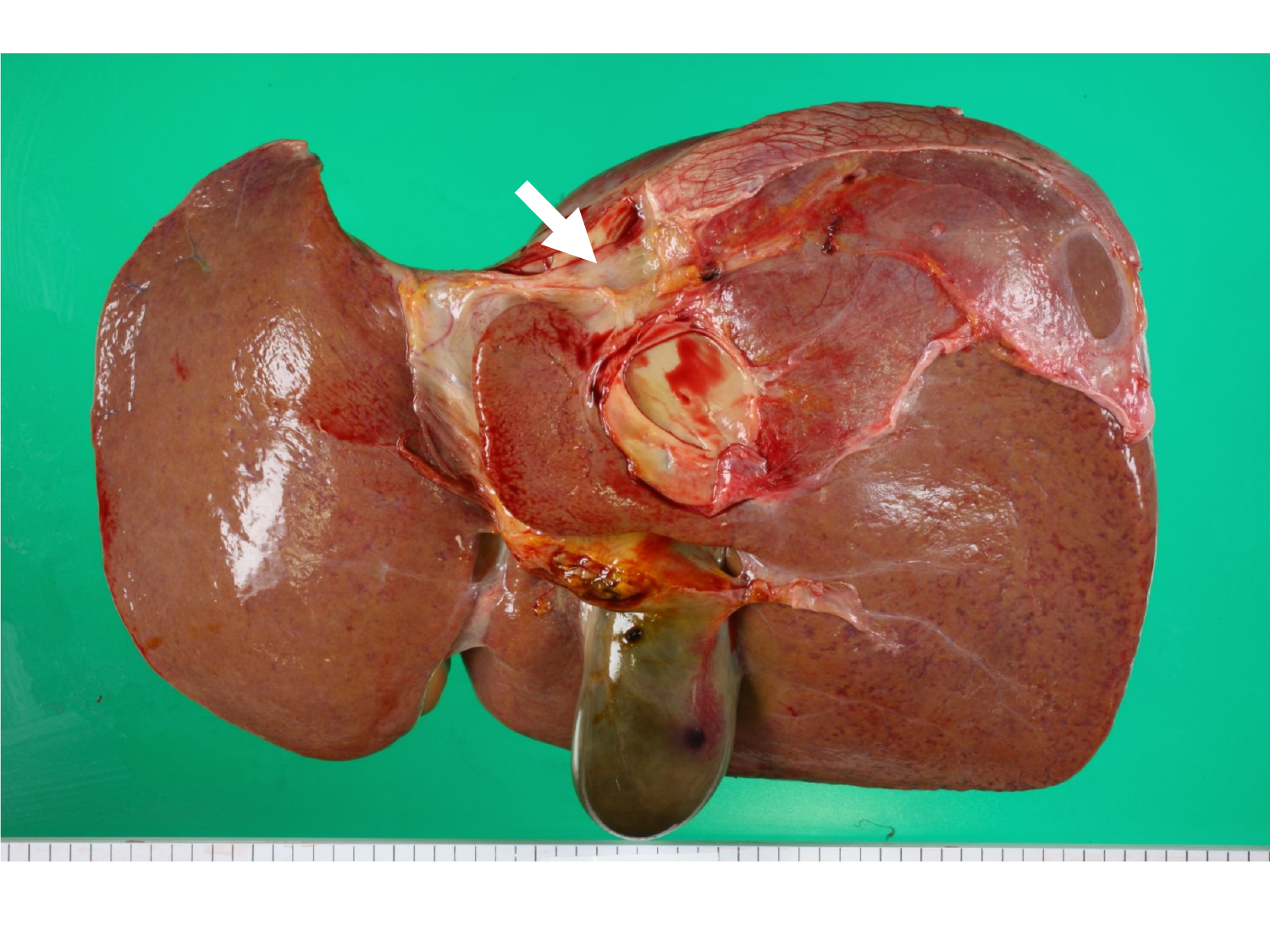

Supplement: Supplementary file 1 — Fig S1A [file CCR3-9-2254-s003.pptx]

## Slide 1
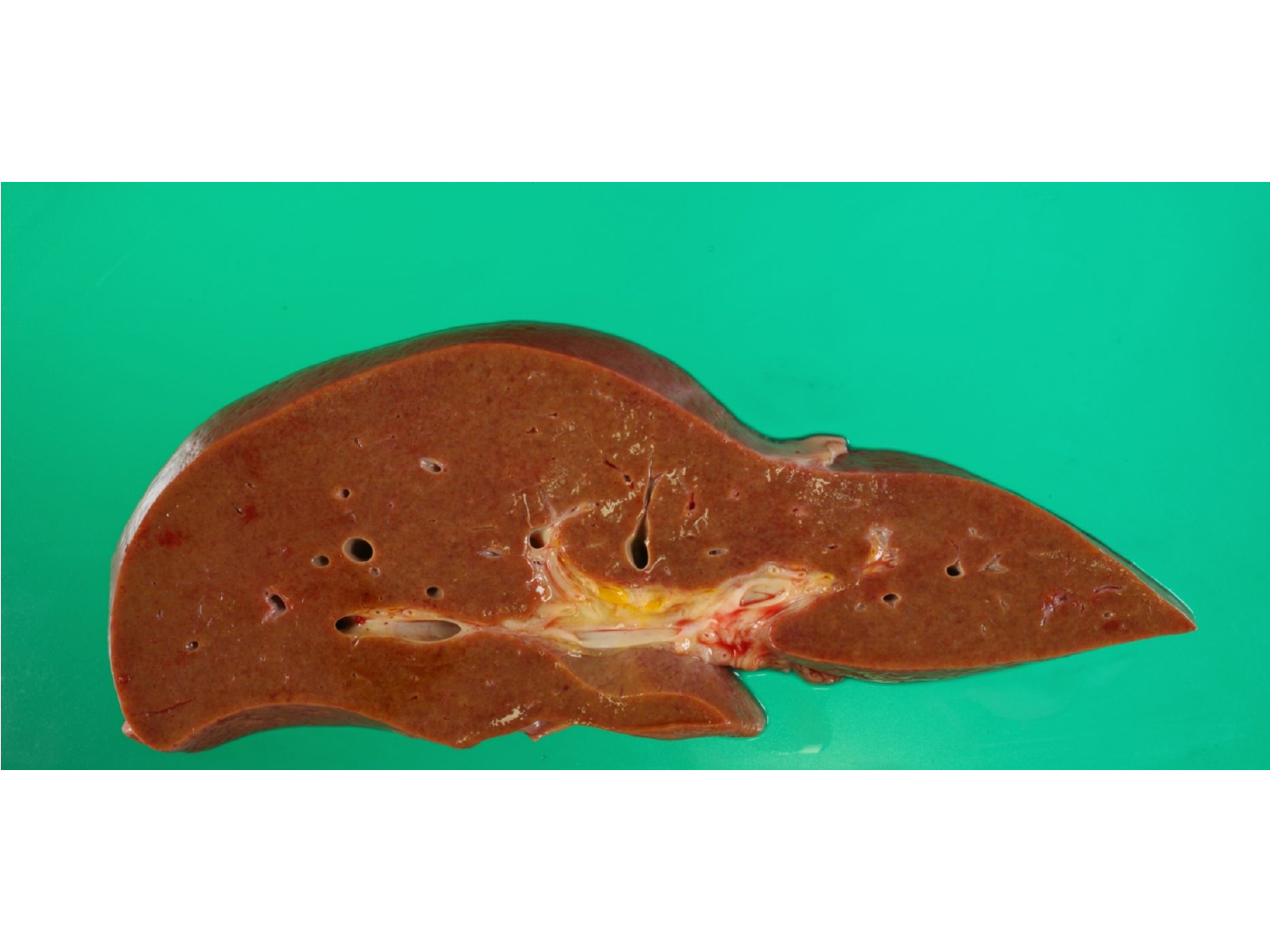

Supplement: Supplementary file 2 — Fig S1B [file CCR3-9-2254-s001.pptx]

## Slide 1
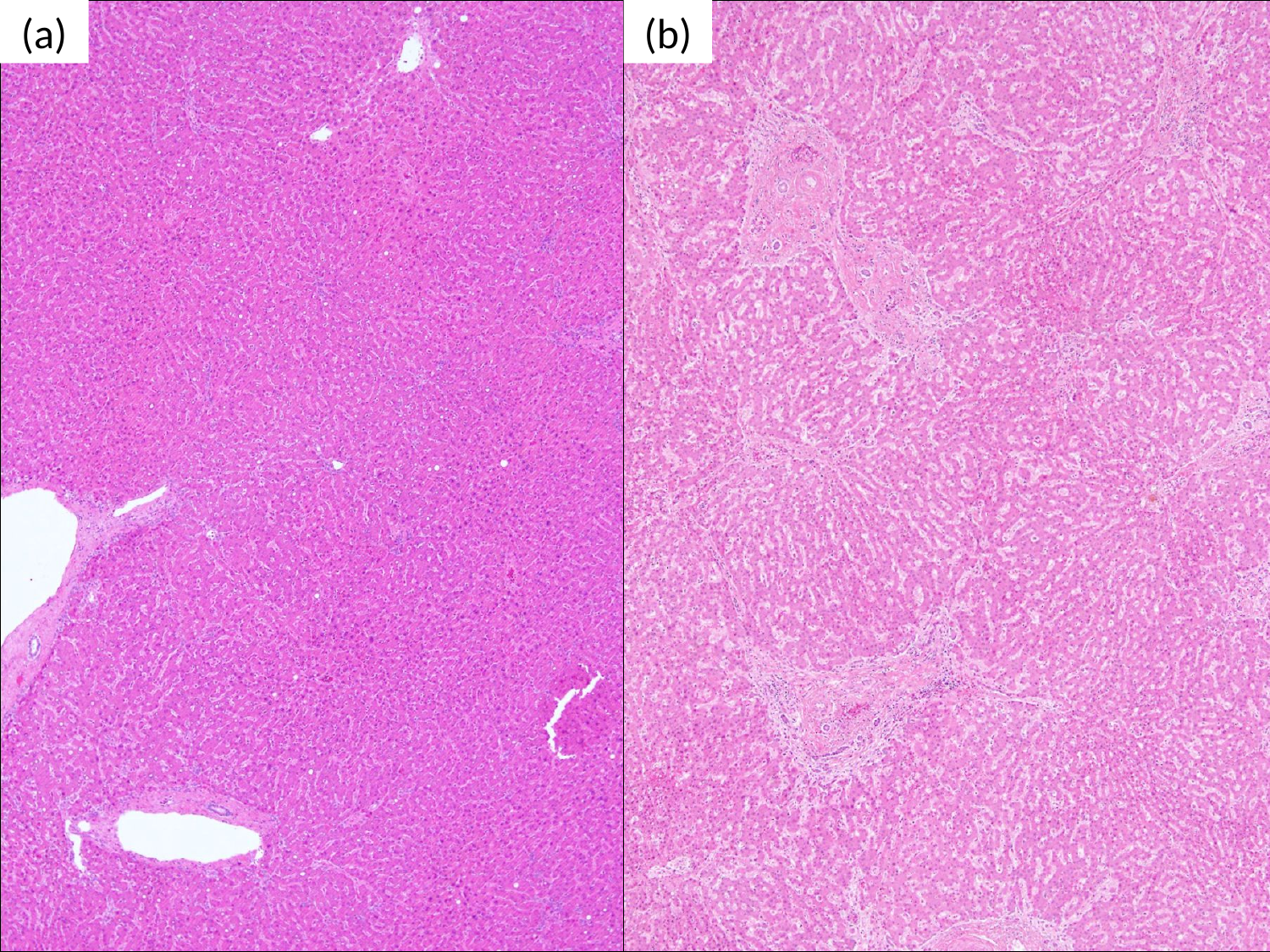

(a)
(b)

Supplement: Supplementary file 3 — Fig S2 [file CCR3-9-2254-s002.pptx]
